# 3-Dimensional Culture Systems for Anti-Cancer Compound Profiling and High-Throughput Screening Reveal Increases in EGFR Inhibitor-Mediated Cytotoxicity Compared to Monolayer Culture Systems

**DOI:** 10.1371/journal.pone.0108283

**Published:** 2014-09-23

**Authors:** Amy L. Howes, Robyn D. Richardson, Darren Finlay, Kristiina Vuori

**Affiliations:** Cancer Center, Sanford-Burnham Medical Research Institute, La Jolla, California, United States of America; University of Sheffield, United Kingdom

## Abstract

3-dimensional (3D) culture models have the potential to bridge the gap between monolayer cell culture and *in vivo* studies. To benefit anti-cancer drug discovery from 3D models, new techniques are needed that enable their use in high-throughput (HT) screening amenable formats. We have established miniaturized 3D culture methods robust enough for automated HT screens. We have applied these methods to evaluate the sensitivity of normal and tumorigenic breast epithelial cell lines against a panel of oncology drugs when cultured as monolayers (2D) and spheroids (3D). We have identified two classes of compounds that exhibit preferential cytotoxicity against cancer cells over normal cells when cultured as 3D spheroids: microtubule-targeting agents and epidermal growth factor receptor (EGFR) inhibitors. Further improving upon our 3D model, superior differentiation of EC50 values in the proof-of-concept screens was obtained by co-culturing the breast cancer cells with normal human fibroblasts and endothelial cells. Further, the selective sensitivity of the cancer cells towards chemotherapeutics was observed in 3D co-culture conditions, rather than as 2D co-culture monolayers, highlighting the importance of 3D cultures. Finally, we examined the putative mechanisms that drive the differing potency displayed by EGFR inhibitors. In summary, our studies establish robust 3D culture models of human cells for HT assessment of tumor cell-selective agents. This methodology is anticipated to provide a useful tool for the study of biological differences within 2D and 3D culture conditions in HT format, and an important platform for novel anti-cancer drug discovery.

## Introduction

The development and utilization of model systems that recapitulate human solid tumor architecture and biology are essential to better understand the pathophysiology of tumor cells, and to aid in the discovery of novel anticancer therapies. As a result, models have been developed to reflect the microenvironment of solid tumors. 3D spheroid cultures can recapitulate cell-cell interactions, cell-matrix interactions, nutrient and oxygen gradients, and cell polarity that is lacking in traditional 2D monolayer culture [1], [2]. 3D cultures also contain heterogeneous zones of proliferating, quiescent, and dying cells, which are likewise found in human tumor tissue and exhibit differing sensitivities to anti-tumor treatments [1], [3]. Thus, 3D cell culture models bring significant value to the drug discovery and development process as a potential practical bridge between traditional *in vitro* monolayer cultures and expensive *in vivo* animal studies [4], [5], [6].

Current treatment for most human cancers includes chemotherapeutic agents that are toxic against dividing cells, frequently resulting in numerous side effects. The approval of molecularly-targeted therapies, such as the protein kinase inhibitors imatinib, gefitinib, and lapatinib, have borne out the promise that agents that specifically target cancer cells rather than all dividing cells result in fewer side effects.

When cytotoxicity studies against cancer cells are performed, cells are typically cultured as a monolayer, where cell-cell contacts and microenvironment signals are lacking and the culture conditions may therefore not reflect the *in vivo* situation for cytotoxicity and/or drug resistance. To circumvent these technical issues, 3D cultures are being formed and analyzed in a variety of interesting formats [7], [8], [9], and co-cultures are being recognized as valuable systems for predicting drug responses *in vivo* for a number of different diseases [10], [11], [12]. A call for complex 3D culture models specifically for breast cancer [13] highlights the importance of the work by Reid *et al.* to measure transcriptional changes in 3D monotypic cultures using high content imaging [14], as well as of our study here where we measure cell viability in high-throughput (HT) amenable 3D co-cultures that demonstrate the usefulness of 3D co-cultures for identifying anti-tumor agents with robust selectivity for tumor cells over normal cells.

Here, we have utilized a modified version of the multi-cellular spheroid “hanging drop” technique [15] and have optimized it in high-density round-bottom plates that have been treated with hydrogels to inhibit cell attachment, enabling formation of single spheroids of reproducible size across several different human cell types. The need for HT-amenable models for cancer research has recently been reviewed [16]. Of the five most prominent methods for generating uniformly-sized spheroids; that is, chitosan hydrogel co-culture, PDMS V-bottom microwells, microfluidic devices, two-layer embryoid bodies, and the multi-well hanging drop (reviewed in [3] and [17]), we reasoned that the multi-well hanging drop model is the most HT-amenable due to cost, meeting liquid handling requirements, and resulting in less cross reactivity with administered compounds. In our studies, we generated 3D cultures of normal and tumorigenic breast epithelial cells suitable for robust cell viability readouts in primary screens and secondary hit confirmation. The spheroids were also found to be amenable to traditional biochemical and cell biological techniques (e.g. immunoblotting and immunostaining), allowing mechanistic studies. Thus, using the same experimental format, we are now able to directly compare the normal cells to tumor cells in 3D culture.

In the present study, we compared the sensitivity of normal and tumor breast epithelial cell lines to the 89 clinically-relevant compounds in the National Cancer Institute’s (NCI) Approved oncology Drug Collection (ADC) [18]. As anticipated, we observed significant cytotoxicity toward both normal and tumor cell lines with a number of the drugs, but also identified microtubule-targeting agents and epidermal growth factor receptor (EGFR) inhibitors as major classes of compounds that exhibited preferential cytotoxicity against tumor cells over normal cells when cultured in 3D. We also generated 3D co-cultures of human breast tumor epithelial cells with stromal and endothelial cells and compared those to 3D co-cultures of the stromal and endothelial cells. The ADC library was again screened using these co-cultures, this time over four concentrations and with enhanced robustness of the assay. The results of these proof-of-concept screens indicated that 3D cultures and co-cultures can be valuable tools for identifying clinically-useful drugs, including molecularly-targeted agents with selectivity for tumor cells over normal cells that have the potential to reduce deleterious side-effects frequently observed with cytotoxic agents.

## Materials and Methods

### Compounds and reagents

The Approved oncology Drug Collection (ADC) was obtained from the National Cancer Institute’s Developmental Therapeutics Program. The collection includes 89 drugs, all maintained at 10 mM in 100% DMSO. For more information, see: http://dtp.nci.nih.gov/branches/dscb/oncology_drugset_explanation.html [18]. Hoechst 33342 was purchased from Invitrogen (H3570), vinblastine and vinorelbine from Sigma-Aldrich (St. Louis, MO), and lapatinib, gefitinib, dasatinib, and Tyrphostin AG1478 from LC Labs (Woburn, MA).

### Cells and Antibodies

MCF-10A cells, BT-474 cells, and human foreskin fibroblasts (Hs.58) were obtained from the ATCC. MCF-10A cells were cultured in 50/50 DMEM/F12, 5% FHS, 1 µg/mL hydrocortisone, 5 µg/mL insulin, 5 ng/mL rhEGF, and penicillin, streptomycin, and glutamine (PSG) supplements. BT-474 cells were cultured in RPMI with 10% FCS and human fibroblasts (HFs) were cultured in DMEM with 10% FCS and PSG. Human umbilical vein endothelial cells (HUVECs) were obtained from Lonza (Walkersville, MD) and cultured in Lonza’s Endothelial Basal Cell Medium (EBM-2) supplemented with EGM-2 singlequots (CC-3124). The CD31 antibody (clone JC70A) was obtained from Dako North America, Inc. (Carpinteria, CA) (#M0823) and used at a 1∶50 dilution, the vimentin antibody (V2122-11E) from US Biological (1∶200 dilution), the β-actin antibody (clone AC-40) from Sigma (1∶5000 dilution for IB) and the HER2 antibody (554299) from BD Biosciences (1∶1000 dilution). The phospho-HER2 antibody (clone 6B12) (1∶100 dilution for IF, 1∶1000 for IB), phospho-EGFR antibody (clone D7A5) (1∶100 dilution for IF, 1∶1000 for IB), and total EGFR antibody (clone C74B9) (1∶1000 for IB) were all purchased from Cell Signaling Technology (Beverly, MA).

### Cell culture conditions in 2D or 3D

MCF-10A, BT-474, human fibroblasts or HUVECs were maintained as monolayer cultures in the media described above. To generate 3D miniature cultures for HTS, cells were seeded at a cell number observed to consistently form a single, uniformly round spheroid. BT-474 cells were seeded at 3000 cells/well (in RPMI+10% FBS+PSG medium) and the MCF-10A cells at 1000 cells per well (in 50/50 DMEM/F12, 5% FHS, 1 µg/mL hydrocortisone, 5 µg/mL insulin, 5 ng/mL rhEGF, PSG) in 96-well round-bottom ultralow attachment plates (Corning #7007). Over a forty-eight hour period spheroids self-assembled from the seeded cells, one spheroid (of ∼250 microns in diameter) per well. Spheroids exceeding 200 microns in diameter were found to have hypoxic cells located on the interior of the spheroid [using the detection reagent hypoxyprobe, NPI Inc. (data not shown)]. The viability of the cells composing the spheroids was checked using ATP as a readout and cells were found to maintain viability for at least five days after plating without having to change the medium or add supplements. For HTS cultures in 2D, common tissue culture-coated, flat-bottom 96-well plates (Corning #3595) were used to grow cells, in the same media and at same cell concentrations as noted above.

For co-cultures in 3D, trypsinized cells were counted and mixed together to be seeded into 96-well round-bottom ultralow attachment plates (Corning #7007). As indicated in the legends, either the total cell number was held at 3000 cells per well, or 3000 BT-474 cells were seeded together with 1500 HFs and/or HUVECs. As we observed with the monotypic spheroids, the co-cultures also spontaneously formed one spheroid per well during the 48-hour incubation period. By analysis of ATP levels, the co-culture spheroids were viable for 5 days without a medium change or addition of supplements. All the co-cultures were maintained in a 1∶1∶1 mixture of RPMI:DMEM:EGM-2 complete media. The 2D co-cultures were plated at the same ratio of cells in the same mixed medium, but in flat bottom 96-well tissue culture plates (Corning #3595). Forty-eight hours after plating, the 2D or 3D cells were used for cytotoxicity assays, screening, or immunostaining as described in subsequent Methods sections.

### Cytotoxicity assay

Cells were plated as described for 2D or 3D culture, and then treated with compounds at the indicated concentration for 48 hours. To quantitate cellular ATP as a measure of viability, 50 µL of CellTiter GLO reagent (Promega) was added to each well. The plates were orbitally shaken for 15 minutes at room temperature to facilitate lysis. In addition, spheroids were triturated six times to promote complete lysis. Well contents were transferred to a white-bottom 96-well plate and read on a Bio-Tek luminescence plate reader.

### Immunofluorescent analysis of spheroid sections

Spheroids were fixed for 1 hour in zinc formalin, washed in PBS, and then frozen in O.C.T. (Takeda) for cryostat sectioning. Ten micron sections were mounted onto slides and stained with appropriate primary antibodies for 1 hour at 37°C. After three thorough washes in 1X TBS+0.2% Tween-20, the slides were incubated with anti-mouse Alexa 488 (Invitrogen) at 1∶500 dilution, anti-rabbit Alexa 594 (Invitrogen) at 1∶750 dilution and Hoechst 33342 at a 1∶10,000 dilution for 1 hour at 37°C. The slides were washed and mounted with Vectashield (Vector Laboratories).

### Microscopy and 3D rendering

Brightfield or fluorescent images (10X) were obtained by an AI Observer Zeiss microscope with Photometrics Coolsnap HQ^2^camera. Confocal (BD CARVII) optical sectioning was performed on z-series captured in 4 micron steps, and then subjected to 3D deconvolution via Autoquant (Media Cybernetics) software, and visualized in Metamorph’s 4D viewer (MDS Analytical Tech).

### Screening protocols

The Approved oncology Drug Collection obtained from the National Cancer Institute was used in the screens. For single concentration point primary screens, the BT-474 cells were seeded at 3000 cells/well (in RPMI+10% FBS+PSG medium) and the MCF-10A cells at 1000 cells per well (in 50/50 DMEM/F12, 5% FHS, 0.5 µg/mL hydrocortisone, 5 µg/mL insulin, 5 ng/mL rhEGF, PSG) in 96-well Corning #3595 (for 2D) or Corning #7007 (for 3D) plates. Dilution plates were made by diluting the compounds 1∶1 with 50% medium/50% DMSO to yield a final concentration of 5 µM per well. Forty-eight hours after plating, the cells were treated with the library of compounds. Following 48 hours of compound incubation, the cells were lysed with 50 µL CellTiterGLO (Promega) reagent with 15 minutes of orbital shaking at room temperature. 75 µL of solution was transferred to Greiner Lumitrac plates and read on a BioTek luminescence plate reader.

For the co-culture screens, serial dilution primary screening was used. The cells were seeded in 96-well plates at 1500 HF+1500 HUVEC cells per well for the human fibroblast and human endothelial cell (HF+HE) co-cultures (in EGM-2 complete medium), or 750 HF+750 HUVEC+1500 BT-474 for the BT-474+HF+HE co-cultures (in 1∶1 RPMI+10% FBS+PSG: EGM-2 complete medium). Cells seeded in Corning #7007 round bottom, ultra-low attachment plates formed a single 3D spheroid per well while cells seeded in Corning #3595 flat bottom, tissue culture-treated plates attached as a 2D monolayer. Following 48 hours of incubation to allow for spheroid formation or cell attachment, the cells were treated using a STAR liquid handler with 1 µL of compound. Here, the Approved oncology Drug Collection was used to prepare master plates via dilution with DMSO using a STAR liquid handler to 10 mM, 1 mM, 100 µM, and 10 µM stock plates to treat the cells with four different final concentrations (100 µM, 10 µM, 1 µM, and 0.1 µM). Lapatinib was added to each dilution plate for a positive control, to yield final in-well concentrations of 10 µM, 1 µM, 0.1 µM, and 0.01 µM. Forty-eight hours later, 50 µL of CellTiterGLO (Promega) reagent was added to each well using a Multidrop Combi. The plates were rocked for 15 minutes to encourage cell lysis. Spheroid lysis was further aided by mixing 100 µl of volume using the STAR liquid handler, and then 75 µl was transferred to a Greiner Lumitrac 96-well plate for luminescence reading on a PE Envision plate reader. For the monolayer plates, 75 µl was transferred to the Greiner Lumitrac plates per well, and also read on the PE Envision.

## Results and Discussion

### Screening of 3D cultures reveals tumor cell selectivity for certain drug classes

**Figure 1:**
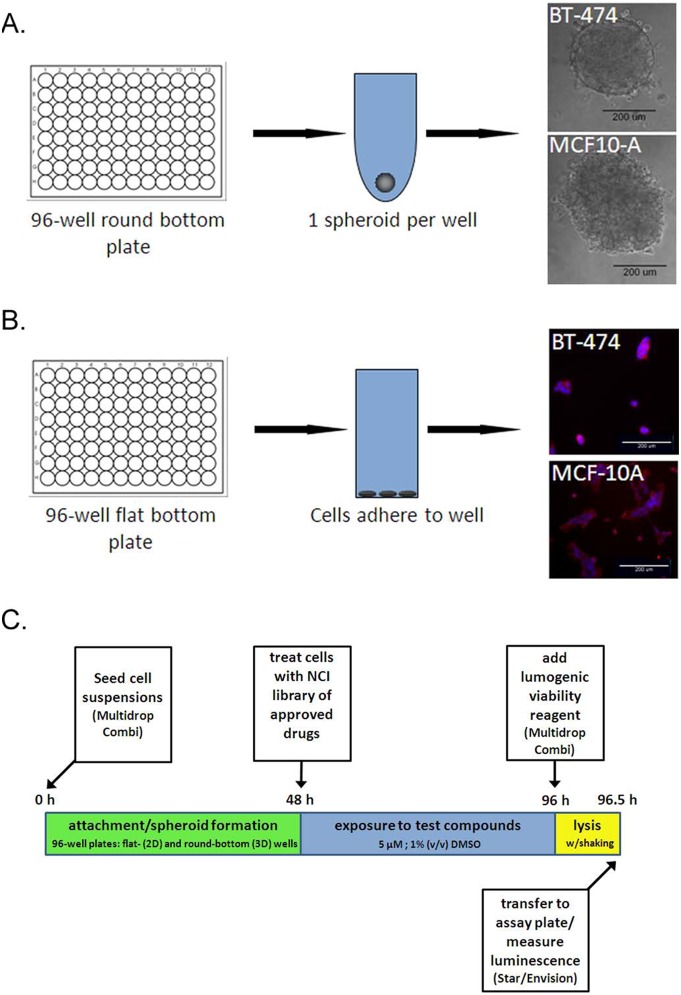
Spheroid and monolayer plating schema and screen design. **A**, one spheroid per well was formed in 96-well round bottom ultra-low attachment plates. Brightfield micrographs of typical BT-474 and MCF-10A spheroids are shown 48 hours after plating, *bar,* 200 microns. **B**, the 2D monolayer cultures were set using the same cell number as in the round bottom plates (3000 cells/well for BT-474 or 1000 cells/well for MCF-10A) in flat bottom, tissue culture-coated 96-well plates. Fluorescence images are shown of typical BT-474 and MCF-10A monolayers 48 hours after plating, stained for actin (red) and DNA (blue), *bar*, 200 microns. **C**, schematic of screen design.

To determine if human cells grown in 3D culture offered a benefit over 2D cultures for identifying anti-tumor compounds with greater selectivity for tumor cells than normal human cells, we optimized the conditions to grow and screen breast epithelial cells in two different multi-well formats. To form 3D spheroids, 96-well U-bottom ultra-low attachment plates were used. This encouraged the cells added to each well to aggregate together and form a single spheroid since they could not adhere to the well surfaces (Figure 1A). 2D monolayers were grown in typical tissue culture-treated, flat-bottom 96-well plates (Figure 1B). Importantly, the breast cancer epithelial cells (BT-474) and non-transformed breast epithelial cells (MCF-10A) were handled and set in the same manner in the two different multi-well formats, minimizing other variables which could affect drug potency. The cells were plated, treated 48 hours later with a final concentration of 5 µM of compound (from NCI’s Approved oncology Drug Collection library), and assayed for ATP content another 48 hours later (Figure 1C). The 48 h time point following plating was chosen based on the time needed for spheroids to form and achieve reproducible compactness (based on spheroid diameter) in each well. The 48 hour time point following drug treatment was identified as the optimal time to observe drug-induced cell death, as medium changes add additional expense to HT assays and longer incubations resulted in untreated cells exhibiting cell death due to exhaustion of medium nutrients and buildup of waste products.

The initial results from this proof-of-concept screen were analyzed by comparing the compounds that produced a positive “hit” (defined as decreasing ATP content by 50% or more compared to vehicle treated cells) in the BT-474 tumor cells compared to the MCF-10A cells. As would be expected for compounds of known cytotoxicities, the hit rate was high for both cell lines, with 29% and 25% of compounds found to be hits in BT-474 and MCF-10A cells, respectively, in 2D, 3D or both culture conditions. Comparing the compounds identified as hits in the two different cell lines in at least in one of the culture conditions, there were few compounds that appeared to be selective for the BT-474 cells over the MCF-10A cells (data not shown). However, as 3D culture conditions are anticipated to more accurately reflect tumor architecture, we focused on those compounds that were hits under both 2D and 3D culture conditions in tumor cells, but only under 2D culture conditions in normal cells, suggesting preferential effects on tumor cells in 3D cultures. These hits included molecularly targeted compounds against EGF receptors, such as gefitinib and lapatinib, and the broad-spectrum kinase inhibitor dasatinib.

**Figure 2:**
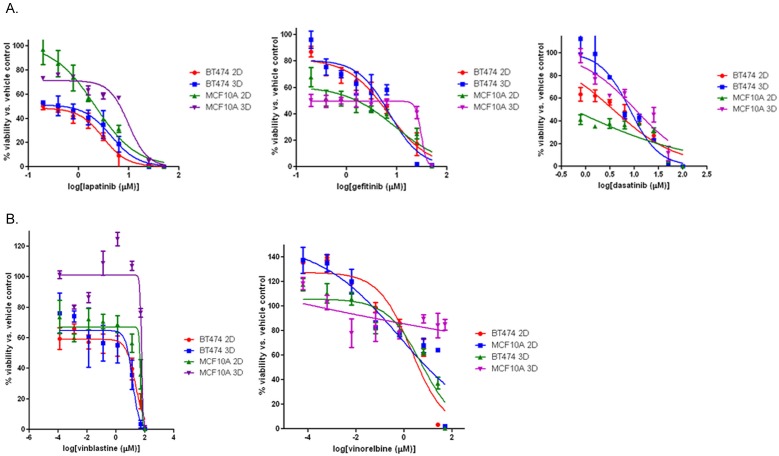
3D vs. 2D screen results in BT-474 and MCF-10A cells. **A**, concentration-response curves were generated from BT-474 or MCF-10A cells set and treated in triplicate with lapatinib, gefitinib, or dasatinib as described in Materials and Methods, and percent viability was calculated from luminescence readings representing ATP content and normalized to vehicle-treated control cells. **B**, concentration-response curves were generated from BT-474 or MCF-10A cells set and treated in triplicate with vinblastine or vinorelbine. All curves are representative data of at least two experiments performed in triplicate.

Dose response experiments were then performed to confirm the results of the primary screen and to identify EC50 values, the concentration at which the compound reduces cell viability by 50%, for the selected compounds on each cell type cultured in 2D and 3D (Table 1, Figure 2). In the MCF-10A cells, the results from the primary screen were confirmed for lapatinib, gefitinib, and dasatinib (Table 1, Figure 1A); under 3D conditions, the MCF-10A cells were relatively insensitive to these compounds (EC50>5 µM). The MCF-10A cells grown as 2D monolayers in turn exhibited EC50 values <5 µM following treatment with lapatinib or dasatinib. As in the primary screen, the BT-474 cells demonstrated sensitivity (EC50<10 µM) under both culture conditions for these compounds (Table 1, Figure 2A), and hence a selectivity for the BT-474 cells over the MCF-10A cells was observed under 3D culture conditions, with the EC50 value for lapatinib <1 µM in BT-474 cells and 9.8 µM in MCF-10A cells (Table 1). The calculated EC50 values for gefitinib and dasatinib were slightly above 5 µM (6–8 µM), which may be due to the different source of compounds used for the confirmatory dose response assays compared to the primary screen (NCI’s ADC library versus LC Labs; NCI library format allows compound use for primary screening only).

**Table 1:** EC50 Values from Secondary Concentration-response Assays on Hits from First Screen.

	lapatinib	gefitinib	dasatinib	vinblastine	vinorelbine
BT-474 2D	<1 µM	6 µM	3.6 µM	21.8 µM	2 µM
BT-474 3D	<1 µM	7 µM	8.1 µM	13.5 µM	5.2 µM
MCF-10A 2D	2.1 µM	9.2 µM	<1 µM	51.6 µM	0.6 µM
MCF-10A 3D	9.8 µM	30 µM	10.5 µM	58 µM	>100 µM

Microtubule targeting agents’ vinblastine and vinorelbine had also scored as positive “hits” in our primary screen, demonstrating slight selectivity for the BT-474 cells over the MCF-10A cells under 3D culture conditions. The results obtained with vinblastine and vinorelbine in the primary screen were not readily confirmed in dose-response studies, however (Table 1, Figure 2B). While vinblastine did show greater selectivity for the BT-474 cells, especially when grown as 3D cultures, the EC50 values we observed were all >5 µM (Table 1, Figure 2B, left panel). In the concentration-response experiments, vinorelbine was indeed a hit in the BT-474 cells but not in the MCF-10A cells cultured in 3D (Table 1, Figure 2B, right panel). However, under 2D culture conditions, we observed EC50 values <5 µM for both cell lines even though we did not identify vinorelbine as a “hit” for MCF-10A cells in the primary screen. To ascertain if any anomalies are due to permeabilization issues we have also confirmed that small fluorescent dye molecules, of similar size to many drugs, can indeed penetrate into the spheroid core (data not shown).

Taken together, our studies demonstrate that it is possible to identify tumor-selective compounds by applying a strategy that utilizes information from both 2D and 3D cytotoxicity screens. Due to the fact that our proof-of-concept study involves a library that consists of known bioactive compounds, the observed differences in sensitivity between normal and cancer epithelial cells may not necessarily be large enough to assure they would be useful in a HTS setting. An unbiased screen with a larger small-molecule library will likely yield different and potentially more informative results. Also, and as demonstrated by our follow-up studies, a traditional HTS which tests compounds at a single concentration can be burdened by false positives and false negatives and requires extensive follow-up testing. A new paradigm, quantitative HTS (qHTS), generates concentration–response curves for test compounds in a single experiment and will likely alleviate these issues and could be a method of choice for a rapid screening of novel anti-cancer compounds [19].

### Co-culture models of normal vs. cancer cells exhibit differing drug sensitivities in 3D vs. 2D

To further improve upon our screening methods, we sought to determine whether human cancer cells cultured as 3D co-cultures with supporting cells such as fibroblasts and endothelial cells could prove more useful in identifying targeted (and, generally, less toxic) compounds. We hypothesized that co-culturing the tumor cells with fibroblasts and endothelial cells could influence the microenvironment of the 3D culture and thus impact drug sensitivity. The tumor microenvironment, including stromal factors such as fibroblasts and endothelial cells, plays an important role in tumor progression and metastasis formation [20]. In normal tissue, epithelial cells are segregated from the stroma by interaction with the basement membrane. In the co-cultures described herein, BT-474 cells interacted directly with fibroblasts and endothelial cells, as can be observed in advanced breast cancers like invasive ductal carcinoma [6], and these culture systems may therefore better mimic the tumor microenvironment of advanced cancers. Many researchers utilize the laminin-rich basement membrane extracted from Engelbreth-Holm-Swarm mouse sarcoma (Matrigel) to promote growth of cells in 3D [3], [5] or in xenograft models, but the expense of this reagent would be prohibitive for a HT-screen and could potentially interfere with certain assay readouts. Thus, our model that utilizes human fibroblasts and endothelial cells to support the growth of tumor epithelial cells in 3D was developed as a cost-effective and practical alternative.

**Figure 3:**
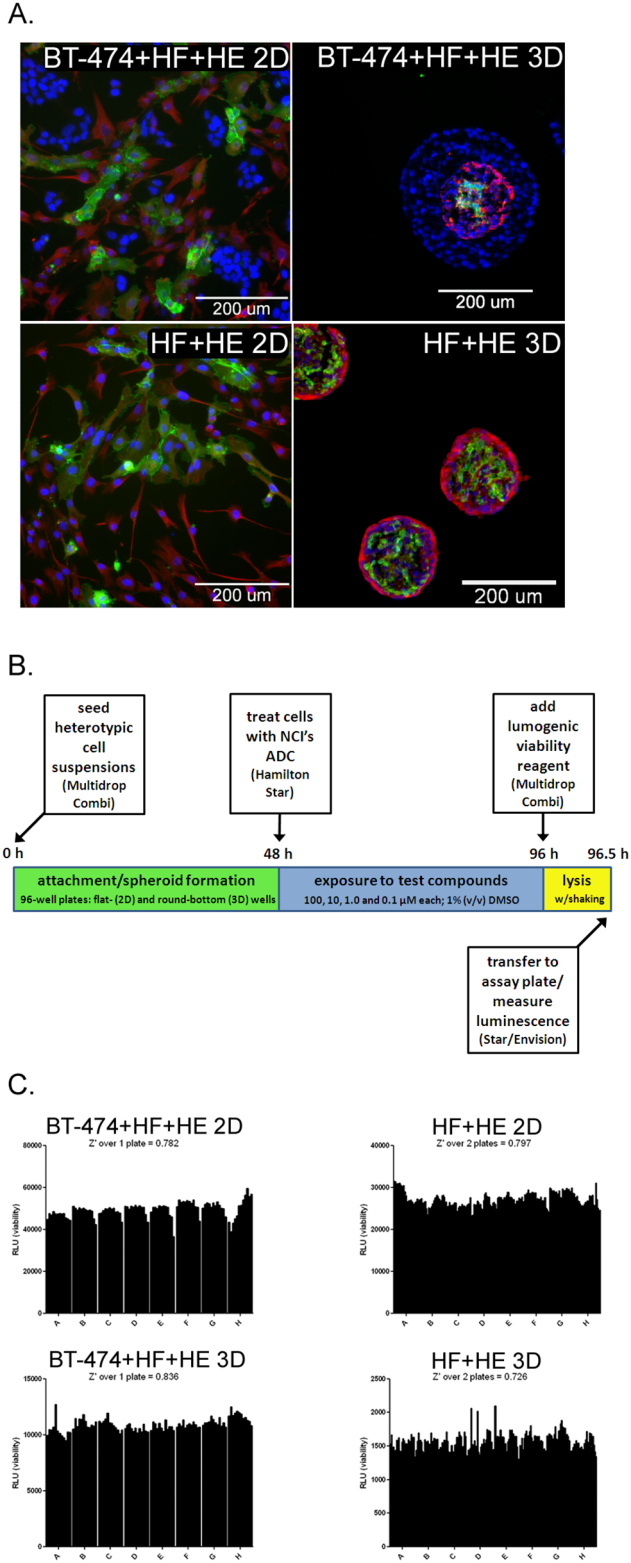
Description of the ADC library screen over four concentrations in 3D vs. 2D co-cultures. **A**, immunofluorescent images of fixed and stained 2D (left panels) and fixed, sectioned, and stained 3D (right panels) co-cultures stained for the endothelial cell marker CD31 (green), the fibroblast-rich protein vimentin (red), and all the cells’ nuclei (blue), *bars,* 200 microns. The top most panels are the BT-474+HF+HE co-cultures, seeded with 1500 BT-474+750 fibroblasts+750 endothelial cells per well. The bottom panels are the HF+HE co-cultures, seeded with 750 fibroblasts+750 endothelial cells. **B**, schematic of the four-concentration screening protocol in BT-474+HF+HE and HF+HE cells cultured in 3D or 2D. **C**, Z’ values generated from one or two 96-well plates set, incubated, and handled as described for the screen for the 2D BT-474+HF+HE cells (top left panel), 2D HF+HE cells (top right panel), 3D BT-474+HF+HE cells (bottom left panel), 3D HF+HE cells (bottom right panel).

We utilized our model system to compare co-cultures containing tumor cells to co-cultures lacking tumor cells. The BT-474 cell line was co-cultured in 2D or 3D (Figure 3A, top left and right, respectively) with normal human fibroblasts and normal human endothelial cells (BT-474+HF+HE). Interestingly, we observed that during co-culture in 3D, the cell types repeatedly self-assembled into a structure where the BT-474 tumor cells surrounded a core of the fibroblasts and endothelial cells. These tumor-cell containing co-cultures were compared to 2D or 3D (Figure 3A, bottom left and right, respectively) co-cultures containing only the normal human fibroblasts and endothelial cells (HF+HE). The CD31-positive endothelial cells appeared as vessel-like formations in the HF+HE cultures similar to what has been previously reported in 3D co-culture with skin fibroblasts and HUVECs [21].

The screen of the BT-474+HF+HE and HF+HE cultures was performed over four different concentrations of the NCI’s ADC library, resulting in final in-well concentrations of 100, 10, 1 and 0.1 µM (Figure 3B). Due to the large number of 96-well plates required to screen at four different concentrations, more automation was utilized than in the previous screen of monotypic BT-474 and MCF-10A cell cultures. To ensure that the assay was robust enough for semi-automated screening, Z’ values were calculated for both co-culture conditions in 2D and in 3D to quantitate the statistical reproducibility of the assay based on the means of the maximal and minimal signals received in each well across multiple plates. This standard calculation is performed to ensure that each well gives off a similar output (chemiluminescence indicating ATP content and thus viability for this screen) such that when compounds are added, a decrease in chemiluminescence is likely to be a result of the compounds’ effect on cell viability and not just variation across the individual wells. We observed Z’ values greater than 0.7 across all co-culture conditions (Figure 3C), thus demonstrating an excellent performance for high-throughput screening.

The results from the co-culture screen were analyzed using a two-tiered approach. First, hits were defined as compounds yielding <50% viability at any of the four concentrations tested, in any of the culture conditions. With such a low level of stringency, 57 of the 89 compounds were found as “hits” in the primary screen (data not shown); again an understandable result based on the nature of the library. Second, concentration-response curves were generated for each compound under each culture condition, and EC50 values were assigned where applicable. We mined the EC50 data to identify compounds that showed greater toxicity toward the cancer cell co-cultures (BT-474+HF+HE) than the normal cell co-cultures (HF+HE) when cultured in 3D, with the rationale that our long-term goal is to use these cultures to identify novel anti-tumor therapeutics with selective toxicity toward tumor tissue, while sparing normal tissue. Twelve compounds exhibited greater selectivity for the 3D BT-474+HF+HE cells than the 3D HF+HE cells (Table 2), including, once again, a number of compounds that target receptor tyrosine kinases or microtubules.

**Table 2:** Screening results comparing normal [HF+HE cells cultured in 2D (“2N”) or 3D (“3N”)] and tumor cell [BT-474+HF+HE cells cultured in 2D (“2T”), 3D (“3T”), or HF+HE cells cultured in 2D (“2N”) or 3D (“3N”)] containing co-cultures in 2D and 3D.

Ixabepilone	IC50 2N<2T<3T<3N	microtubule stabilizer
vinorelbine	IC50 2N<2T<3T<3N	antimitotic
paclitaxel	IC50 2N<2T<3T<3N	microtubule stabilizer
rapamycin	IC50 2N<2T = 3T<3N	mTOR inhibitor
azacitidine	IC50 2N<2T<3T<3N	antimetabolite
dasatinib	IC50 2N<2T<3T<3N	BCR-ABL/src inhibitor
vinblastine	IC50 2N<2T<3T<3N	microtubule inhibitor
vincristine	IC50 2N<2T<3T<3N	microtubule inhibitor
taxotere	IC50 2N<2T<3T<<3N	microtubule stabilizer
nilotinib	IC50 2N<2T<3T<3N	BCR-ABL inhibitor
gefitinib	IC50 3T<<2N = 2T = 3N	EGFR inhibitor (TK domain)
lapatinib	IC50 3T<<3N<2N<2T	HER2 and EGFR inhibitor

**Figure 4:**
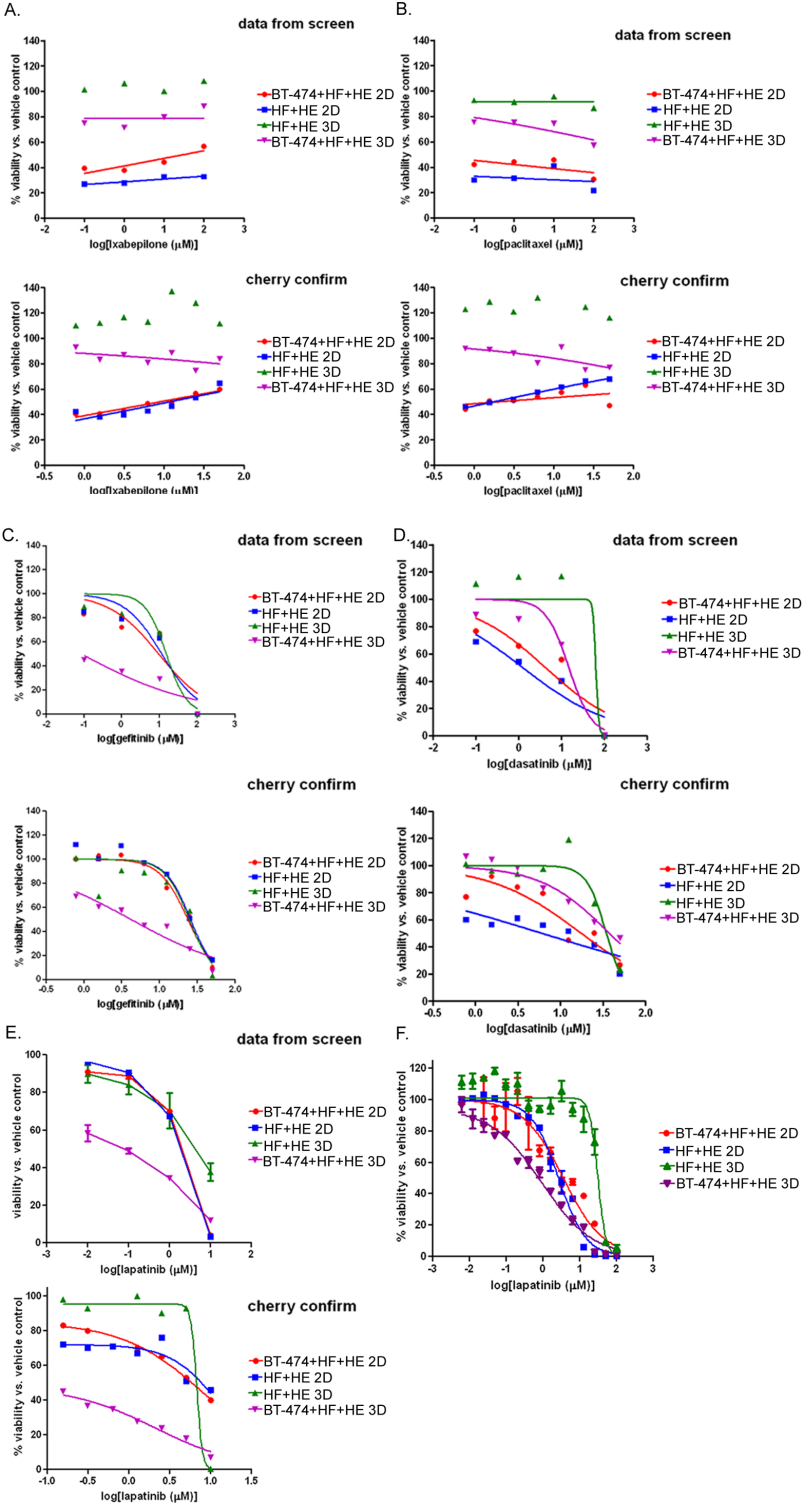
Screening hits and secondary confirmation concentration response assays. **A–E**, concentration-response curves generated from the data obtained in the screen (top panels) compared to those curves generated from cherry-picked wells containing ixabepilone (A), paclitaxel (B), gefitinib (C) or dasatinib (D) or lapatinib (E). For all curves, the cells were plated as described in Fig. 3A and treated as described in 3B. **F**, Concentration-response curve generated by treating 2D BT-474+HF+HE (“2T”), 3D BT-474+HF+HE (“3T”), 2D HF+HE (“2N”), or 3D HF+HE (“3N”) co-cultures with concentrations of lapatinib ranging over 5 logs for 48 hours. Cell viability was assessed using the CellTiterGLO assay, as described for the screens, and normalized to vehicle control. Experiment was performed in triplicate, *bars*, standard deviation.

We followed up the primary co-culture screen with a secondary concentration-response assay to confirm the twelve hits listed in Table 2. The compounds were cherry-picked from the master library plates prepared for the screen, and used to perform concentration-response experiments over the same time course (48 hours of treatment). The microtubule-targeting compounds exhibited a rather flat curve over the concentrations tested, but the relative potency of the drugs on the different co-cultures was verified in the secondary assays (Figure 4). All six of the microtubule-targeting compounds were confirmed in secondary assays as exhibiting greater toxicity towards tumor cell co-cultures (BT-474+HF+HE) than normal co-cultures (HF+HE) when cultured as 3D spheroids. The original curves from the screen and the curves from the secondary assay using cherry-picked compounds are shown for the compounds ixabepilone and paclitaxel (Figures 4A and 4B). Importantly, the 3D HF+HE co-cultures appeared insensitive to the microtubule inhibitors unlike the 3D BT-474+HF+HE co-cultures. The microtubule-targeting agents were more potent in both of the 2D co-culture conditions, but lacked the specificity for the tumor-cell containing co-cultures in 2D. Microtubule inhibitors used in the clinic induce undesirable side-effects such as nausea and neutropenia, so although it was reassuring to see these effective anti-tumor compounds as hits in the screen, follow-up studies were subsequently performed with molecularly-targeted compounds that may exhibit fewer side effects, in line with the big picture goals of our studies.

The molecularly-targeted agents gefitinib, dasatinib, and lapatinib exhibited more typical sigmoidal curves over the range of concentrations tested in both the original screen and secondary assays (Figures 4C–F). Because lapatinib was used as a positive control on each of the assay plates in the primary screen, there were enough wells to calculate standard deviations in our small-scale proof-of-concept screen (Figure 4E, top panel). We observed that 3D HF+HE and BT-474+HF+HE co-cultures had strikingly different sensitivities to lapatinib, so to quantify an EC50 value, a 15-point concentration response assay was performed in triplicate over five logs of lapatinib concentrations (Figure 4F). The calculated EC50 values clearly indicated that under 2D culture the sensitivity to lapatinib was similar (BT-474+HF+HE = 3.5 µM compared to HF+HE = 2.7 µM), while the same cells cultured in 3D differed in sensitivity by about 30-fold (EC50 for BT-474+HF+HE = 0.9 µM compared to HF+HE = 30 µM).

We examined the contribution of the different cell types to the observed sensitivity to lapatinib under 3D culture conditions. Based on the data in Figure 4F, we hypothesized that the cancer cells were the cells increasingly losing viability in the heterogeneous 3D co-cultures. We indeed observed that BT-474 spheroids were more sensitive to lapatinib than the BT-474+HF+HE co-cultures, consistent with the hypothesis that the fibroblast and endothelial cells maintained viability in the presence of lapatinib. Spheroids composed of HF or HF+HE were 10- and 100- fold less sensitive than the BT-474 cancer cell spheroids, respectively (Figure S1A). Fluorescent images of control or lapatinib-treated spheroids which were composed of BT-474 (unstained)+HF (red)+HE (green) cells also suggested that the BT-474 cells were preferentially losing viability and dissociating from the spheroid co-culture (Figure S1B). Although EGFR inhibitors were rationally designed based on the biochemistry of EGFR dependent tumors, our methodology suggests they would also have been detected in a rigorous 3D screen. This is indeed the main point of our study, i.e. that the model or system is capable of detecting relevant drugs rather than an investigation of the drugs themselves.

Taken together, our studies utilizing co-culture methods establish a methodology for HT screening in 2D and 3D. Importantly, our pilot screen further highlights the discriminatory power of 3D culture conditions, and suggests that co-culture models mimicking tumor cell-stroma interactions may represent an important future direction for drug discovery and development.

### 3D cultures exhibit reduced EGFR and HER2 activation

**Figure 5:**
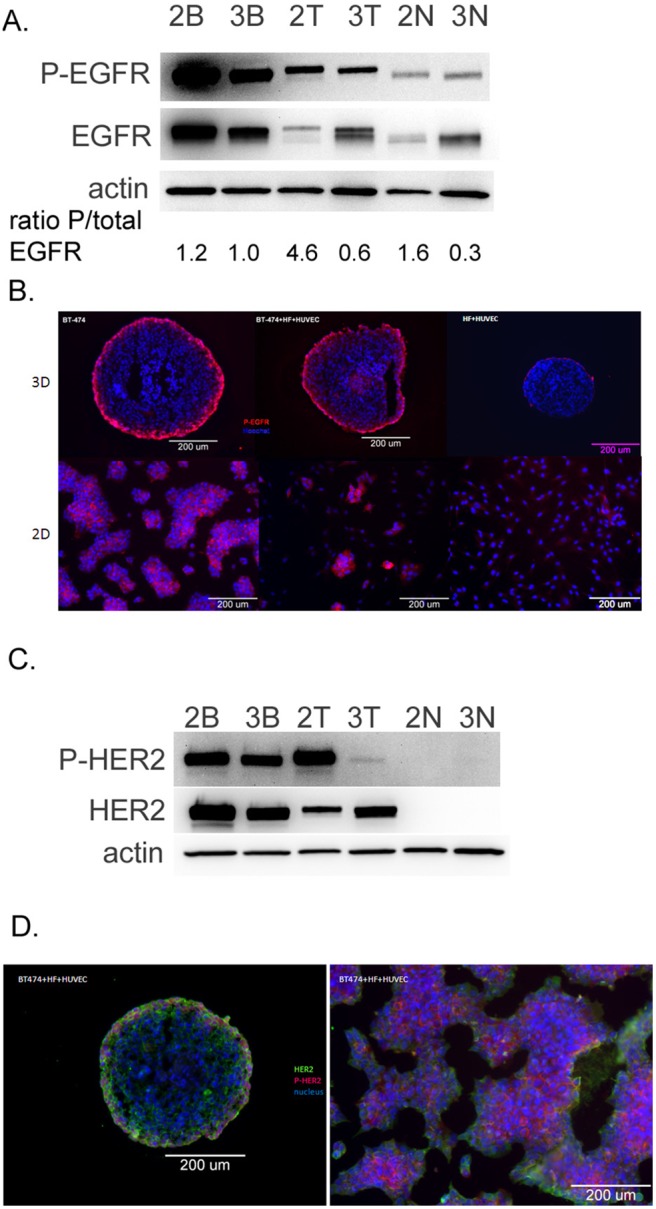
EGFR and HER2 receptor expression and phosphorylation in 3D vs. 2D cultures. BT-474 cultures were seeded at 3000 cells/well on round-bottom (for 3D cultures, labeled 3B in figure) or flat-bottom (for 2D cultures, labeled 2B in figure) dishes. Co-cultures were seeded (3000 BT-474+1500 fibroblast+1500 endothelial, or 1500 fibroblast+1500 endothelial) on round-bottom (for 3D) or flat-bottom (for 2D) 96-well dishes. Seventy-two hours later the cells were lysed and harvested for immunoblot analysis, or fixed and stained for immunostaining. **A**, immunoblots against the phosphorylated EGF receptor, total EGF receptor, and actin in tumor cells alone (BT-474 cells) cultured in 2D (lane “2B”), 3D (lane “3B”), tumor cell-containing co-cultures (BT-474+HF+HE cells) cultured in 2D (lane “2T”), 3D (lane “3T”), or cultures lacking tumor cells (aka “normal” co-cultures containing HF+HE cells) cultured in 2D (lane “2N”) or 3D (lane “3N”). **B**, immunostaining of EGFR phosphorylation (red) and nuclei (blue) in BT-474 cells (left panels), BT-474+HF+HE cells (middle panels), or HF+HE cells (right panels) cultured in 3D (top panels) or 2D (bottom panels), *bars*, 200 microns. **C**, immunoblots against the phosphorylated HER2 receptor, total HER2 receptor, and actin in BT-474 cells cultured in 2D (“2B”), 3D (“3B”), or BT-474+HF+HE cells cultured in 2D (“2T”), 3D (“3T”), or HF+HE cells cultured in 2D (“2N”) or 3D (“3N”). Independent immunoblot analysis was performed twice and ratios represent mean values of the replicates. **D**, immunostaining of HER2 phosphorylation (red), total HER2 protein (green), and nuclei (blue) in BT-474+HF+HE cells cultured in 3D (left panel) or BT-474 cells cultured in 2D (right panel), *bars*, 200 microns. Representative images are shown of three independent experiments.

The increased sensitivities of cells grown in 3D rather than 2D culture conditions to compounds targeting EGF receptors suggest that the receptor expression or signaling may be altered under the different culture conditions. Specifically, molecular targets for lapatinib and gefitinib include EGFR and HER2 [22], suggesting that HER2 and/or EGFR expression or signaling may be differentially affected. Indeed, previous studies have demonstrated differential EGFR and HER2 receptor expression, activation, and downstream signaling in 3D cultures compared to 2D cultures and concomitant differential sensitivity towards HER2 inhibitors in several human tumor cell lines [23], [24], [25]. To analyze EGF receptor expression and activation in our model system, we performed immunoblotting in samples from lysed 2D or 3D cultures. We observed that EGFR phosphorylation was reduced in BT-474 cells cultured in 3D compared to 2D (Figure 5A). Furthermore, we observed that total EGFR protein expression levels were increased in the 3D BT-474+HF+HE and HF+HE lysates compared to 2D culture conditions while phosphorylation levels remained similar (Figure 5A), suggesting that a relative decrease in EGFR protein phosphorylation also took place in 3D versus 2D co-cultures. This is consistent with studies utilizing a colon cancer cell model by Luca *et al*. [25]. These authors showed that wild type K-Ras colon cancer cells induced to form 3D spheroids by growth in a laminin-rich extracellular matrix demonstrated decreased sensitivity to the EGF receptor inhibitor AG-1478 with a concomitant reduction in EGFR levels.

Important spatial information can be lost during immunoblotting of 3D spheroid protein lysates, so further examination of the EGF receptor expression and phosphorylation was examined by immunostaining. We observed that the EGFR phosphorylation in the 3D cultures and co-cultures was confined to the outermost cells of the culture, as observed in immunofluorescence in fixed 2D and fixed and sectioned 3D samples (Figure 5B). Of note, the anti-CD31 and anti-vimentin immunostainings shown in Figure 1 are performed under similar conditions, and the stainings clearly reach the inner parts of the spheroid indicating the phospho-EGFR staining in the outermost cells only is not artifactual. While changes were observed in EGF receptor expression and/or activation in 2D vs. 3D cultures, the trend appeared to be similar in tumor and normal cells.

We then turned our attention to HER2 expression and activation. As expected, the HF+HE co-cultures lacked any HER2 expression or phosphorylation (Figure 5C). BT-474 cells are derived from a breast cancer patient with HER2 amplification [26], and robust HER2 phosphorylation was observed in these cells, especially under 2D co-culture conditions. Remarkably, HER2 phosphorylation was significantly weaker in 3D co-culture conditions, despite the fact that total HER2 protein levels were found to be elevated in 3D co-cultures compared to 2D co-cultures (Figure 5C). As was the case with EGFR, we observed HER2 phosphorylation only in the outermost cells of the spheroid, while BT-474 cells cultured in 2D exhibited HER2 phosphorylation in most cells throughout the culture (Figure 5D).

The striking effect of chemical HER2 inhibition prompted studies to knockdown the HER2 expression by genetic means using lentivirus-mediated shRNA gene-silencing in BT-474 cells; however BT474 clones that exhibited no HER2 expression by immunoblotting failed to proliferate and could not be used for mechanistic studies in 2D versus 3D cultures (data not shown).

We speculate that in response to sustained receptor activation under 2D culture conditions, the receptor is endocytosed and degraded in an attempt to down-regulate the signal, resulting in an overall decrease in total receptor expression relative to the 3D culture. However, future work will be required to address this mechanism in detail. Also of note, as hypoxia has been found in multicellular 3D cultures similar to ours [1], it is reasonable to suspect that it may play a role in the decreases observed in phosphorylated HER2, but in experiments designed to grow the spheroids under hypoxic conditions to test the effects in the outermost cells, viability was lost (data not shown).

Taken together, we observe differences in the spatial organization, intensity and protein levels of key signaling molecules that are targets for molecular therapies in 2D versus 3D cultures. While the mechanistic insight of this observation needs to be further explored, this finding may explain the differences we observe with regard to lapatinib and gefitinib sensitivity between 2D and 3D co-cultures.

In summary, we have developed 3D culture models that successfully identify clinically useful anti-tumor agents with specificity for human tumor cells over normal human cells. These models are robust and reproducible for HTS studies and can also be used to explore mechanisms of action using traditional cell biological and biochemical techniques. Thus, we expect these 3D model systems to be useful for further mechanistic studies to address the problems of acquired resistance to molecularly targeted therapies (as have been described, for example, in patients treated with HER2 inhibitors), or to investigate new synergistic therapeutic combinations [27]. Additionally, and as put forth by others [6], development of effective and durable cancer treatment strategies is likely to require a mechanistic understanding of the influence of the microenvironment on the therapy response, and we expect the co-culture methodologies developed here to enable this goal in high-throughput manner.

## Supporting Information

Figure S1 Cancer cells are preferentially targeted by the EGFR inhibitors. **A**, cell viability assay of 3D cancer cell cultures showing increased sensitivity to EGFR inhibition as compared to HF cells cultured as 3D spheroids. All spheroids were cultured and treated as in Figure 3A–B. Figure represents 2 experiments performed in duplicate and table utilizes EC50 values from Figure 3 as a direct comparison. **B**, immunofluorescent and brightfield overlay images of BT-474 cells (3 k cells, brightfield), HEs (1 k cells, green) and HFs (1 k cells, red) cultured as spheroids for 48 h before addition of vehicle (0.1% DMSO, left panel) or 10 µM Lapatinib for 48 h (right panel).
(PSD)
